# Could Radiographs Be More Helpful in the Removal of an Intraorbital Foreign Body Than a Surgical Navigation System? A Neurosurgical Case Report

**DOI:** 10.7759/cureus.59062

**Published:** 2024-04-26

**Authors:** Mustafa Ali, Yavor Enchev

**Affiliations:** 1 Department of Neurosurgery and ENT Diseases, Division of Neurosurgery, Medical University of Varna, Varna, BGR

**Keywords:** neuro-ophtalmology, orbital trauma, surgical navigation system, skull radiographs, intraorbital foreign body

## Abstract

Intraorbital foreign body (IOFB) is a vision-threatening condition that requires careful management. IOFB can manifest clinically from asymptomatic up to severe inflammation and blindness. Diagnosis and treatment are determined by the nature of the IOFB. The type, location, and complications related to the IOFB are taken into consideration when planning the surgery. Here, we report the case of a male in his 20s who was admitted to our clinic with a computed tomography (CT) scan which verified the presence of an IOFB. The patient underwent surgery and the IOFB was removed. Using a surgical navigation system (SNS), it was difficult to pinpoint the IOFB precisely during surgery. We took radiographs with a C-arm to improve our orientation and locate the IOFB. The patient recovered uneventfully, and no issues were noticed one month following surgery. This case report highlights the selection of treatment methods and demonstrates when radiographs can be more helpful than an SNS in the removal of the IOFB.

## Introduction

Intraorbital foreign body (IOFB) is a vision-threatening condition that requires a careful approach, diagnosis, and treatment because of the possibility of disabling the patient. Mostly, IOFB is associated with work-related injuries, and males predominate [1]. The clinical presentation of IOFB may vary from asymptomatic to severe inflammation and blindness [2]. The nature of the IOFBs is very important, which determines the diagnosis and treatment; they can be classified as metallic (steel), nonmetallic inorganic (glass), or organic (wood or vegetable) [3]. Surgical planning is based on the nature of the IOFB, localization (anterior or posterior orbit), and foreign body-related complications (such as inflammation, optic nerve injury or compression, extraocular muscle involvement, and orbital fracture) [3,4]. Intraoperatively, a microscope, surgical navigation system (SNS), C-arm, and ultrasound (US) can be used to facilitate the removal of the IOFB [1,5,6].

## Case presentation

Patient history

A 29-year-old man presented to the emergency service with a very small perforating wound in the central part of the lower eyelid. While working with a hammer, he felt something hit his right eye. IOFB was suspected, and the patient was referred to an ophthalmologist. The ophthalmological examination revealed edema of the lower eyelid of the right eye with a perforating wound. There was subconjunctival hemorrhage with a visual acuity (VA) of 20/20 and intraocular pressure of 30.6 mmHg. The mobility of the eye was preserved but painful. Similar to fundoscopy, the anterior and posterior chambers as well as the cornea were normal. The wound of the eyelid was treated, and the IOFB was diagnosed with a computed tomography (CT) scan. Because of the extrabulbar localization of the IOFB, it was recommended that it be removed by a neurosurgeon. One month later, the patient was admitted to our Clinic of Neurosurgery.

Patient examination

Upon admission to our clinic, the patient had a scar on the lower eyelid of the right eye. VA was 20/20. There was no evidence of persistent subconjunctival hemorrhage. The mobility of the eye was preserved, and the patient was without any complaints.

**Figure 1:**
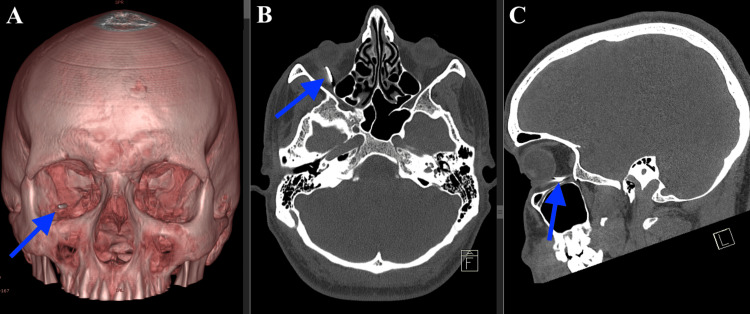
CT scan of the head CT: Computed tomography; IOFB: intraorbital foreign body; 3D: three-dimensional (A) 3D reconstruction of the CT scan showing the IOFB (blue arrow). (B) Axial plane of the CT scan showing the IOFB (blue arrow). (C) Sagittal plane of the CT scan showing the IOFB (blue arrow)

The CT scan showed an extraconal metallic foreign body within the right orbit without conclusive evidence of inferior rectus muscle and periorbital damage (Figure 1).

Surgery

**Figure 2:**
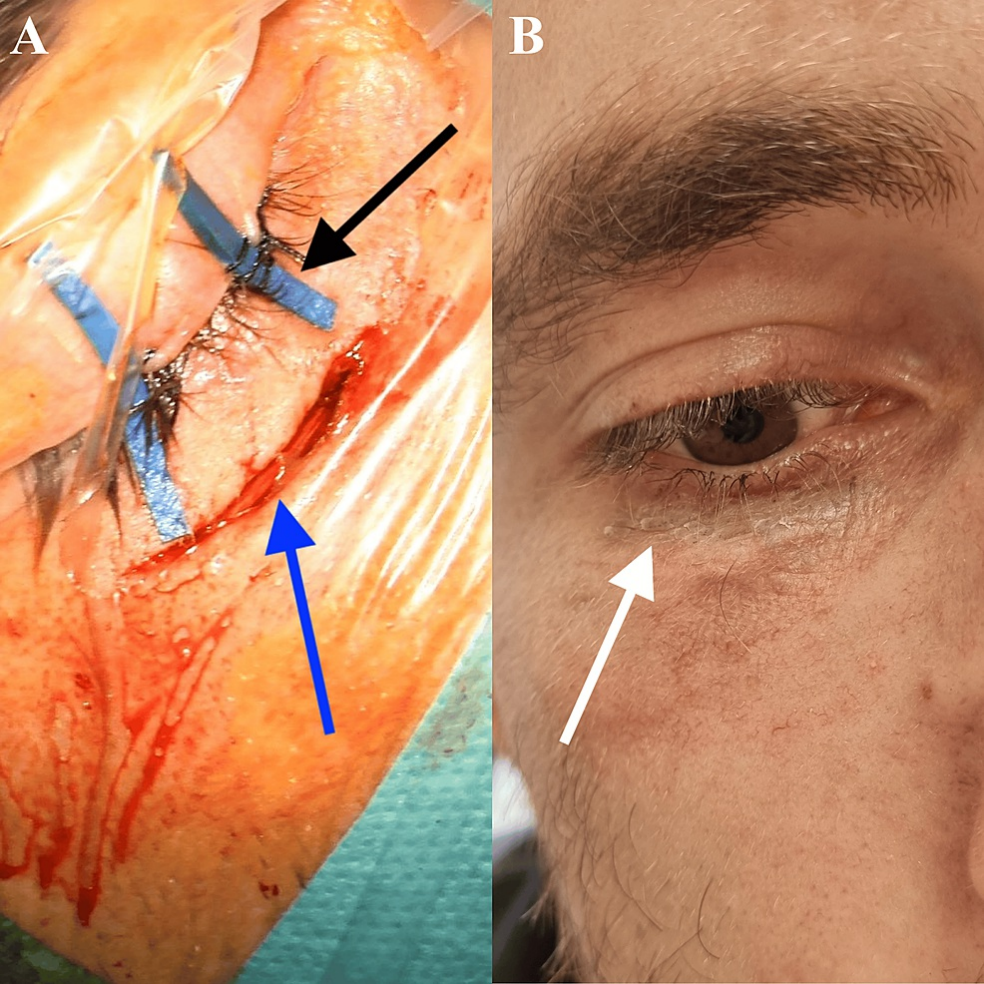
Skin incision and postoperative scar (A) Tarsorrhaphy (black arrow) and infraorbital skin incision (blue arrow). (B) Postoperative scar (white arrow)

**Figure 3:**
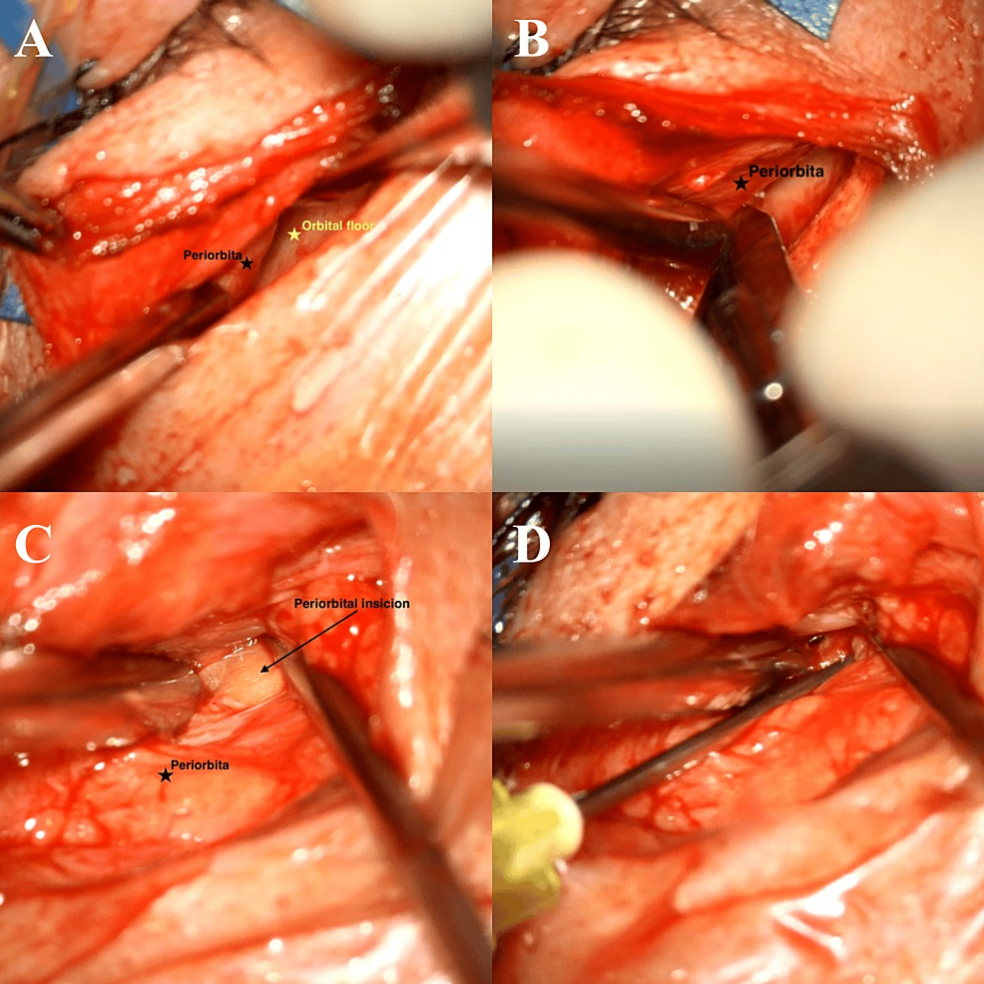
Intraoperative view under а microscope (A) Dissection along the course of the orbital floor using a microsurgical technique. (B) Periorbital incision. (C) Dissection in the extraconal space. (D) Blunt syringe needle used as marker intraoperatively

**Figure 4:**
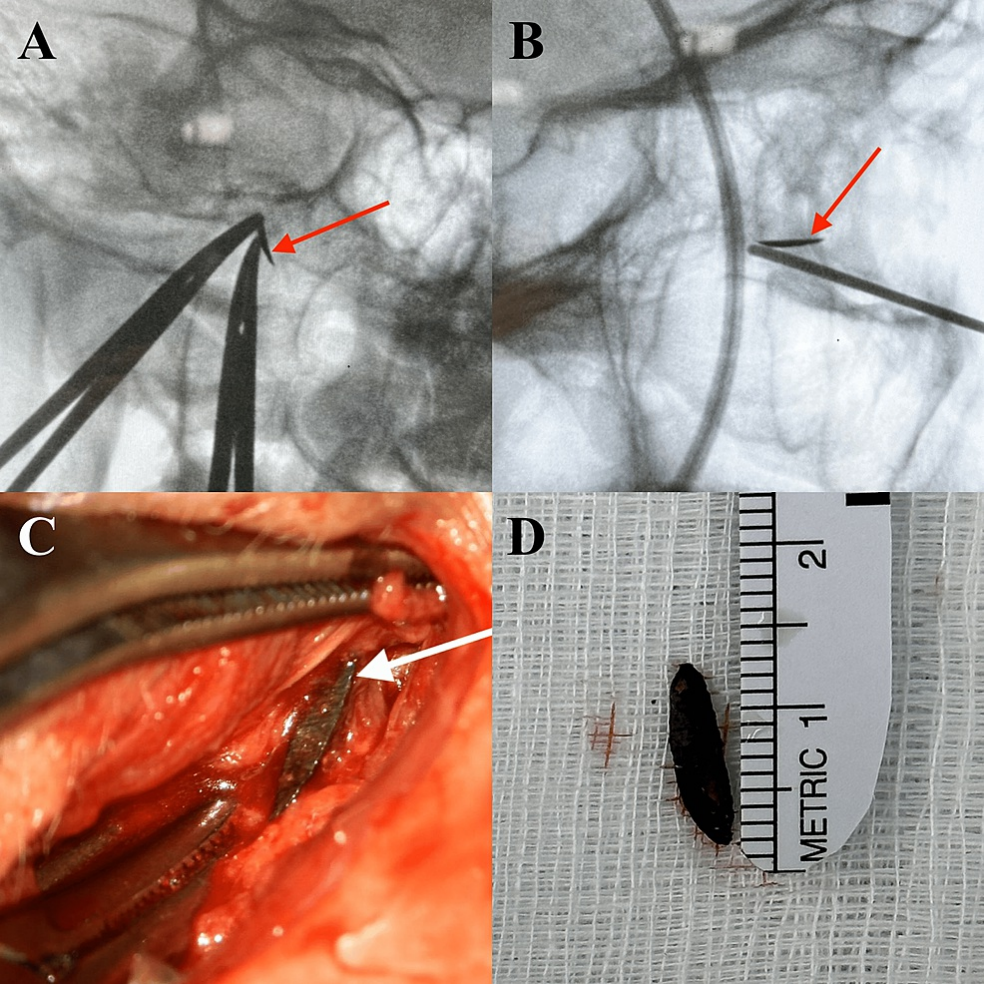
Radiographs and the IOFB IOFB: Intraorbital foreign body (A) Radiograph with mosquito forceps used as markers. (B) Radiograph with a blunt syringe needle used as marker. (C) Mosquito forceps (markers) intraoperatively and the foreign body (white arrow). (D) The foreign body

**Figure 5:**
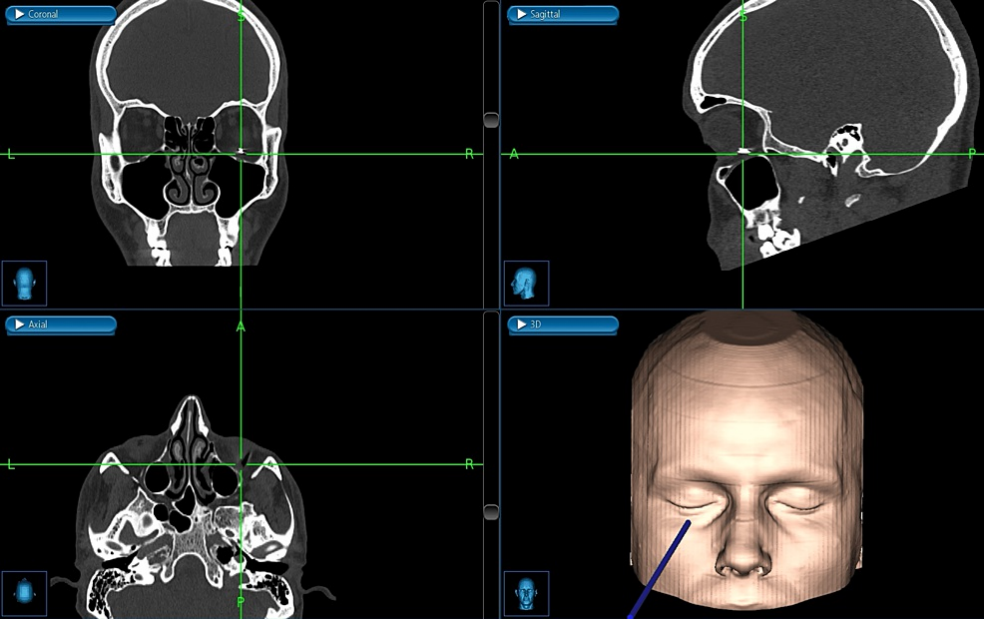
Intraoperative surgical navigation system

Under general anesthesia, tarsorrhaphy was performed, followed by an infraorbital skin incision (between the thin eyelid skin and the thicker cheek skin) (Figure 2A). After dissection along the course of the orbital floor using the microsurgical technique (Figure 3A), the level of the IOFB was reached using the SNS (Figure 5). The periorbita was not damaged, and a periorbital incision was followed by dissection (Figure 3B-3C). Precisely locating the IOFB was difficult with SNS (Figure 5). C-arm radiographs were taken in two projections using markers for better orientation. This helped to locate the IOFB, which was completely covered by connective tissue and situated entirely in the extraconal space (Figure 4A-4C). Hemostatic matrix application was followed by skin sutures and bandage over the entire eye.

Postoperative period

The tarsorrhaphy and bandage were removed on the second postoperative day. Postoperatively VA was 20/20, and he had conjunctival hyperemia and also reported double vision when looking up.

The patient recovered well, and one month after surgery, there were no complaints. The double vision had disappeared, and the surgical incision had healed completely (Figure 2B).

## Discussion

The clinical presentation of IOFBs is variable [2]. Usually, metallic and nonmetallic inorganic IOFBs are well-tolerated and may not have any symptoms or signs if they do not damage the ocular structures, whereas organic IOFBs are poorly tolerated and initiate intense inflammation [3].

Routinely, plain radiography is the first option for the diagnosis of IOFB. It could detect metallic IOFBs in 69% to 90%, but the detection rate for organic material (such as wood) is from 0% to 15% [3]. CT scan is recommended to identify the proper position and visualize the surrounding orbital tissues [3]. The image quality of CT will be limited by metallic IOFBs [5]. Magnetic resonance imaging (MRI) should be performed if the IOFB is small and/or organic [3]. In cases of suspicion of metallic IOFB, MRI should be avoided [2]. US is useful when IOFB cannot be visualized with a CT scan (e.g., glass or plastic) [1]. 

Treatment is based on the nature of IOFBs, localization, and foreign body-related complications [3,4]. Indications for surgery in cases with IOFB are a sharp end, signs of infection, proptosis, restricted motility, palpable orbital mass, optic nerve compression, abscess, suspicion of organic material, fistula formation, or when adjacent structures are compromised [7]. Posteriorly located IOFBs without any clinical features should be left in place because of serious complications, as opposed to anteriorly located IOFBs, which can be removed [3]. Iron-containing IOFBs can lead to siderosis, which is characterized by heterochromia, pigmentation of the anterior chamber structures, mydriasis, and increased intraocular pressure [1,2]. This condition threatens the vision and requires IOFB removal [1,5]. Inert substances (glass, plastic, gold, and silver) are usually well-tolerated and, if asymptomatic, can be managed with periodic follow-ups [1]. Because there was the potential for bulb damage and siderosis in our case, surgery was indicated. 

For fresh traumatic IOFB, the primary wound is the best skin incision and approach [5]. We used an infraorbital approach because the trauma in our patient was a month earlier (Figure 2A). Intraoperative localization of IOFBs may be technically challenging; in this situation, SNS, US, and C-arm can be used [1,5,6]. Intraoperative US may be helpful in the removal of nonmetallic foreign bodies (glass or plastic) because it offers the advantage of real-time imaging from different angles and detecting intraocular pathologies (retinal/choroidal detachment or vitreous hemorrhage) during the surgery [1]. The localization of IOFB can change if there is excessive soft tissue handling or mobilization before accurate localization during surgery [5]. As a result, the accuracy of SNS can be compromised because the system uses preoperative images (CT scan, MRI). In our case, precisely locating the IOFB was difficult with SNS. For better orientation, we took radiographs in two projections with a C-arm and used blunt syringe needles and mosquito forceps as markers to find the IOFB within the surrounding tissues (Figures 3D, 4A-4C).

## Conclusions

The location and nature of the foreign body are important factors to consider when managing an IOFB. When the IOFB is not fixed to a bony structure, finding it with an SNS can be very difficult, as in this case. In these cases, radiographs taken in two projections using blunt syringe needles and forceps as markers may be helpful. With this technique, we can create a real-time coordinate system that makes it easier to pinpoint the IOFB.
